# Autogenous bone augmentation from the zygomatic alveolar crest: a volumetric retrospective analysis in the maxilla

**DOI:** 10.1186/s40729-020-00258-y

**Published:** 2020-10-15

**Authors:** Irina Kuster, Livia Osterwalder, Silvio Valdec, Bernd Stadlinger, Maximilian E. H. Wagner, Martin Rücker, Dominique Bichsel

**Affiliations:** 1 Clinic of Cranio-Maxillofacial and Oral Surgery, Centre of Dental Medicine, University of Zurich, Plattenstrasse 11, 8032 Zurich, Switzerland; 2 Clinic of Cranio-Maxillofacial and Oral Surgery, University Hospital Zurich, Rämistrasse 100, 8091 Zurich, Switzerland

**Keywords:** Autogenous bone augmentation, Zygomatic alveolar crest, Autogenous bone grafting, Bone volume

## Abstract

**Background:**

Autogenous bone augmentation is the gold standard for the treatment of extended bone defects prior to implantation. Bone augmentation from the zygomatic crest is a valuable option with several advantages, but the current literature for this treatment is scant. The aim of this study was to evaluate the increase in bone volume after locoregional bone augmentation using autogenous bone from the zygomatic alveolar crest as well as the complications and success rate.

**Results:**

Analysis of the augmented bone volume in seven patients showed a maximum volume gain of 0.97 cm^3^. An average of 0.54 cm^3^ of autogenous bone (SD 0.24 cm^3^; median: 0.54 cm^3^) was augmented. Implantation following bone augmentation was possible in all cases. Complications occurred in three patients.

**Conclusion:**

The zygomatic alveolar crest is a valuable donor site for autogenous alveolar onlay grafting in a locoregional area such as the maxillary front. Low donor site morbidity, good access, and its suitable convexity make it a beneficial choice for autogenous bone augmentation.

## Background

Sufficient bone volume at the desired implantation site is a requirement for long-term osseointegration of implants [1]. Vertical and transversal alveolar ridge atrophy following tooth loss leads to reduced aesthetics and to a more difficult treatment process [2, 3]. Local bone defects in the alveolar ridge can be augmented with a high success rate, few complications, and a high survival rate of implants [4]. The aim of autogenous bone augmentation is to gain vital hard tissue for functional and aesthetic rehabilitation. The treatment options for alveolar ridge augmentation include guided bone regeneration, distraction osteogenesis, alveolar ridge expansion, and autogenous bone transplantation. Autogenous bone transplantation continues to be the gold standard for two-step procedures: autogenous bone augmentation followed by implant insertion [5]. Success rates of autogenous bone grafting exceed 95% [5]. Multiple intraoral donor sites exist for harvesting autogenous bone grafts. Main donor sites are the ramus mandibulae, the retromolar region, and the symphysis area [6]. In particular, when impacted third molars are present, simultaneous bone harvesting in the retromolar region can be performed or the tooth itself can serve as a potential graft material for alveolar ridge augmentation [7, 8]. Apart from autogenous bone or teeth, and the application of xenogeneic augmentation materials, synthetic-based bone substitutes can be used, which have the advantage of having an unlimited supply. Furthermore, allografts are another alternative for the reconstruction of bone defects [9].

In addition to classical techniques of autogenous bone harvest, the zygomatic alveolar crest has been described as a donor site [2, 10, 11]. Gellrich et al. [2], Held et al. [10], and Kainulainen et al. [11] recommended the zygomatic alveolar crest as an intraoral donor site for local bone augmentation. Sakkas et al. [12] considered the augmentation from the zygomatic alveolar crest as a secure procedure to re-establish optimal conditions of the alveolar crest after small- to medium-sized alveolar defects. Especially in the upper jaw, bone grafts harvested from the zygomatic alveolar crest are particularly suitable due to the locoregional harvesting site. Furthermore, it offers easy access and good visibility as well as a suitable morphology due to its convexity [5, 11].

The aim of this study was to evaluate the increase in volume after bone augmentation with grafts taken from the zygomatic alveolar crest, as well as the assessment of complications.

## Methods

### Aim, design, and setting of the study

This retrospective study analysed patients treated with an autogenous bone graft harvested from the zygomatic alveolar crest between January 2015 and June 2018 in the Clinic of Cranio-Maxillofacial and Oral Surgery, University Hospital Zurich, University of Zurich, Zurich, Switzerland, in order to evaluate the increase in bone volume achieved by the augmentation as well as the success rate (possible implantation after augmentation) and complications. The donor area of the retromolar region is the first-line treatment in our clinic for advanced alveolar ridge augmentation. The zygomatic alveolar crest is part of a broader spectrum of possible donor sites. Therefore, between 2015 and 2018, only sixteen patients were treated with a bone block augmentation from the zygomatic alveolar crest.

### Patients

Only those patients with a pre- and post-operative 3D radiological image of the same image type (digital volume tomography or computer tomography image) were included. The post-operative image had to show the augmented bone area without implants having been placed. Pre-operative images ranged from 2 weeks to 3 months prior to augmentation, whereas post-operative images ranged from 2 to 3 months post-augmentation. The augmented area served as the region of interest.

Exclusion criteria included the use of only two-dimensional (2D) imaging or post-operative imaging post-implant placement and medical history of head and neck radiotherapy, anti-resorptive therapy or local/generalised periodontitis.

### Surgical procedure

All patients underwent a two-stage surgical approach by the same surgeon, whereby onlay bone grafting was the first step. The approach was carried out under local anaesthesia. Prior to the surgical intervention, all patients were instructed to rinse their mouths with chlorhexidine 0.2% for 1 min.

**Fig. 1:**
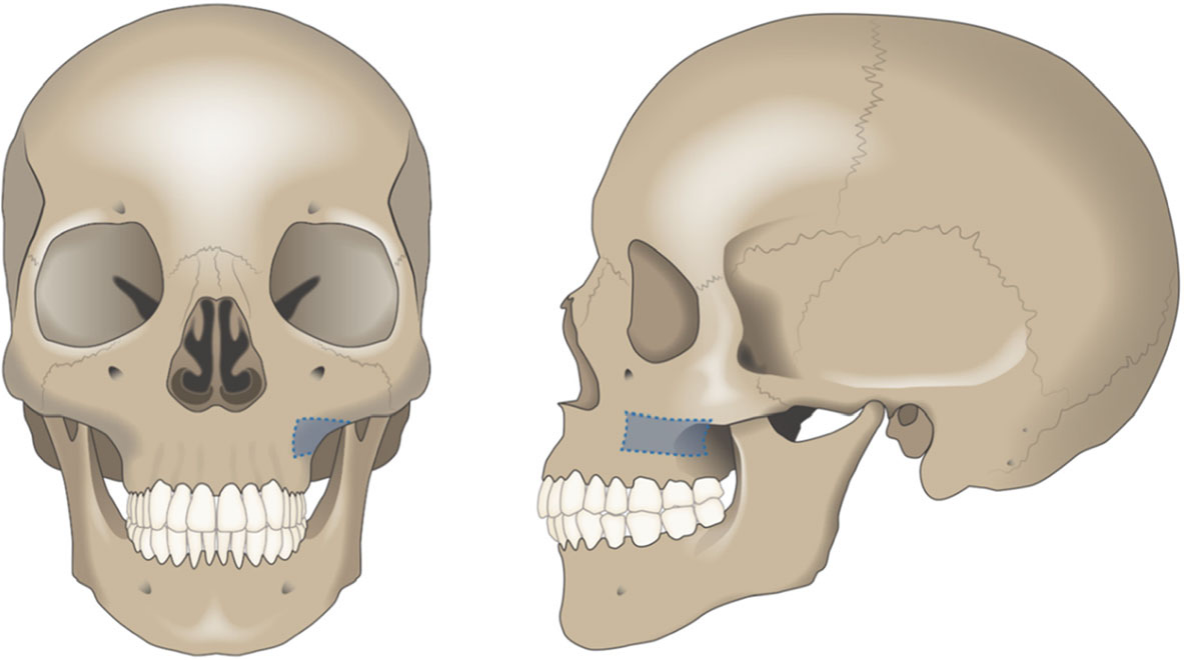
The frontal and sagittal view of the area of the zygomatic alveolar crest where the bone grafts were taken

**Fig. 2:**
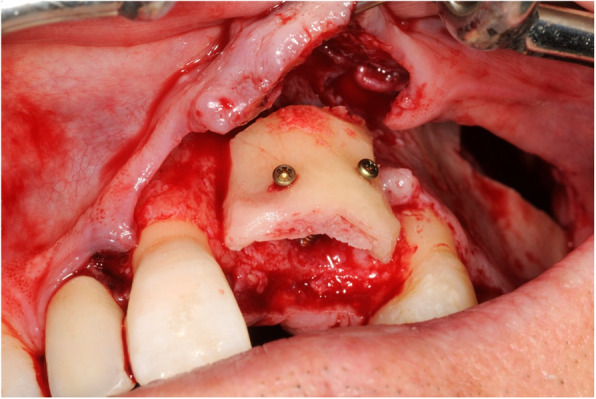
The harvested bone block at the recipient site of patient 3

At the recipient site, sulcular and crestal incisions together with release incisions were performed. For access to the zygomatic alveolar crest, the marginal incisions were expanded to the maxillary posterior region (depending on the recipient site) with a vertical relief incision. In some cases, a mucosal vestibular incision was performed for access. Preparation of a mucoperiostal flap and its mobilisation under haemostatic control enabled full view and surgical access to the recipient site and ipsilateral donor site, from which the cortical bone was harvested (Fig. 1). Piezosurgery (Mectron®; Carasco-GE, Loreto, Italy) was used for bone extraction at the zygomatic alveolar crest and simultaneous soft tissue (Schneiderian membrane) preservation. When needed, additional autogenous bone was harvested with a bone scraper (Safescraper® Twist; CGM SpA, Divisione Medicale META, Reggio Emilia, Italy) at the facial maxillary sinus wall. The cortical bone block was adapted to the recipient area and fixated by two miniscrews (smartDrive®; KLS Martin Group, Tuttlingen, Germany) (Fig. 2). If multiple areas needed to be augmented, the harvested cortical bone graft was divided in two or three parts. In case of extended dimensions of the harvested graft, some parts were crushed in a bone mill. The remaining gap was filled with the crushed bone material or the obtained bone of the bone scraper and then covered by a Bio-Gide® membrane (Geistlich Pharma AG, Wolhusen, Switzerland). Periosteal incision and adaptation of the mucoperiostal flap free of tension ensured optimal coverage of the surgical area.

The post-operative protocol included antibiotic medication for 10 days (amoxicillin/clavulanic acid 1 g, twice a day; clindamycin 600 mg, thrice a day, in case of penicillin allergy), mouth rinse twice a day (chlorhexidine gluconate 0.2%), and no use of dental prostheses for approximately 4 weeks. The patients were strictly advised not to smoke during the healing period or brush their teeth in the surgical area for 2 weeks. The follow-up appointment was 7 days after surgery, and suture removal was scheduled 14 days post-operatively at the earliest.

### Volume calculation

As primary outcome, post-operative volume changes in bone tissue in the grafted area were calculated by computer-assistance with iPlan® ENT 3.0 (Brainlab AG, Munich, Germany). Three-dimensional imaging was performed with different cone beam-computer tomography (CBCT) scanners, namely 3D Accuitomo 170 (J Morita Europe GmbH) and KaVo 3D eXam (KaVo Dental GmbH). The increase in bone volume of the augmentation area was calculated prior to implant insertion.

**Fig. 3:**
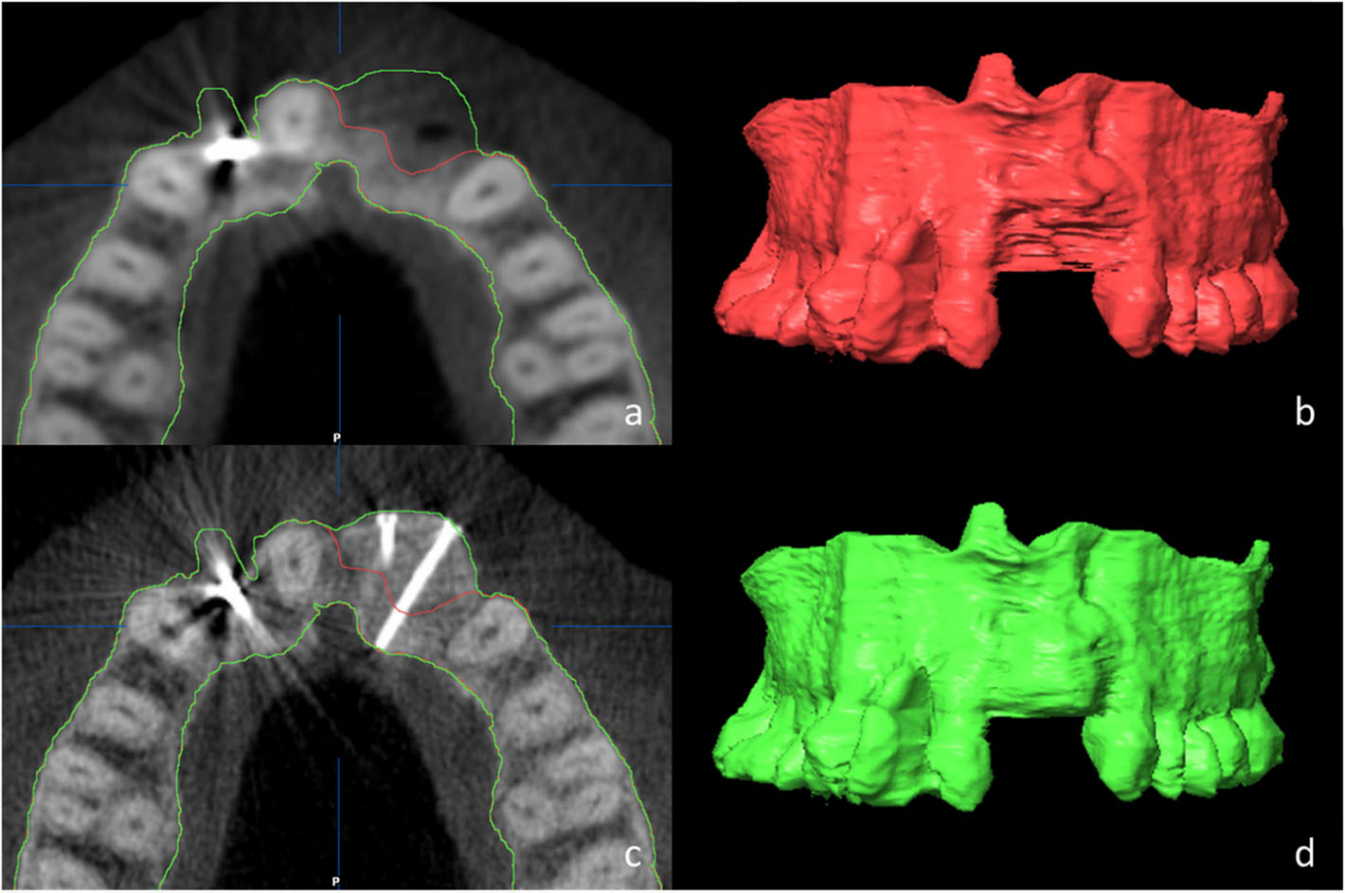
**a**, **c** The axial view of the pre- and post-operative radiological situation with the corresponding borders marked in red (pre-operative situation) and green (post-operative situation). **b**, **d** The corresponding segmented models

An atlas-based auto-segmentation of the upper jaw was performed with iPlan® ENT 3.0 (Brainlab AG, Munich, Germany) with the pre- and post-operative three-dimensional images of each patient and detailed adjustments of the proposed borders were carried out manually (smart brush) as described by Wagner et al. [13]. After merging the pre- and post-operative images, the difference in volume was calculated (Fig. 3).

### Secondary outcomes

Complications and success of the procedure were analysed as secondary outcomes. Success of the treatment was defined as the possibility of implantation following bone augmentation. Complications were defined as wound dehiscence and haematoma formation; failure was defined as complete loss of the grafted bone block.

### Statistical analysis

Data was collected in Excel (Microsoft Corp., Redmond, USA). A descriptive analysis was performed. Mean values, standard deviation, and medians were calculated.

## Results

This study included seven patients (female, 5; male, 2) aged between 17 and 81 years (mean, 53 years; median, 57 years), who met the aforementioned inclusion and exclusion criteria and had no additional severe co-morbidities. They suffered from missing maxillary front teeth or missing teeth in the posterior region. Tooth loss was caused by facial trauma in six patients (tooth avulsion, intrusion, and root fractures) and insufficient bridge restoration due to root fracture in one patient. They all showed a vestibular horizontal and vertical reduction of the alveolar crest. All patients received autogenous bone augmentation grafted from the zygomatic alveolar crest. Five patients received augmentation in the anterior maxilla and two patients in the anterior and posterior maxilla (regions 21–25 and 15–16); the donor site in these cases was always ipsilateral. In four cases of bone augmentation in the maxillary front, bone from the left zygomatic crest was used. Five patients received one bone block, one patient two bone blocks, and another patient three bone blocks, depending on the extent of bone defect. The mean healing period between bone augmentation and implant insertion lasted 103 days.

Of the seven patients, one was a smoker, who was strictly advised not to smoke after surgery.

### Increase in bone volume

The grafted bone volume was evaluated from 50 to 92 days (mean, 75 days) after the augmentation procedure through three-dimensional (3D) radiological imaging (patient 1, 91 days; patient 2, 57 days; patient 3, 92 days; patient 4, 78 days; patient 5, 78 days; patient 6, 50 days; patient 7, 78 days). As shown in Table 1, bone augmentation succeeded in all seven patients, with a maximum increase of 0.97 cm^3^ (range, 0.17–0.97 cm^3^) and a mean of 0.54 cm^3^ (SD, 0.24 cm^3^; median, 0.54 cm^3^). At the time of the post-operative radiological imaging, the soft tissues were irritation-free and fully recovered, except in patient 6 where there was a dehiscence of soft tissues.

**Table 1:** Increase in bone volume

Patient	Pre-operative bone volume (cm^**3**^)	Post-operative bone volume (cm^**3**^)	Increase in bone volume (cm^**3**^)	Radiological evaluation (post-augmentation) (days)
1	**29.76**	**29.93**	**0.17**	**91**
2	**26.00**	**26.71**	**0.71**	**57**
3	**30.81**	**31.35**	**0.54**	**92**
4	**50.16**	**50.64**	**0.48**	**78**
5	**24.02**	**24.37**	**0.35**	**78**
6	**23.53**	**24.50**	**0.97**	**50**
7	**18.50**	**19.08**	**0.58**	**78**

Table 1 shows the pre- and post-operative volume as well as the amount of augmented bone per patient. Patients 6 and 7 received 3 and 2 bone blocks, respectively, from the same donor site. Patients 1 to 5 were each augmented with one bone graft.

### Success rate

**Fig. 4:**
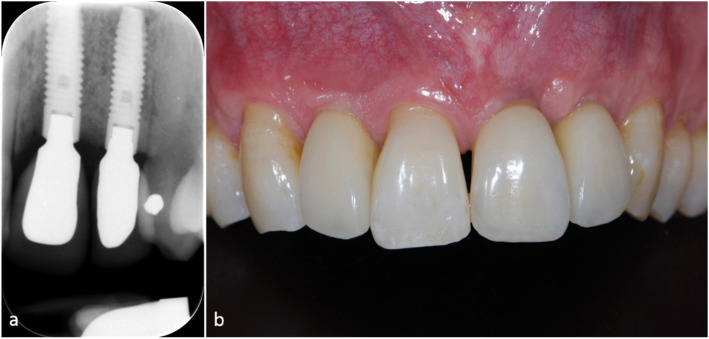
Reference: The apical dental x-ray 10 months after implantation (**a**) and the consecutive prosthetic reconstruction (**b**), Courtesy of N. Nänni & D. Thoma, Clinic of Reconstructive Dentistry, University of Zurich

Implant placement into the augmented site was possible in all cases. In one case, additional bone substitute material was inserted at the time of implantation due to a dehiscence of the implant; in three cases, little cortical bone was harvested with a bone scraper to add more bone material. Figure 4 shows the radiological and clinical situation of patient 3 after implantation with the inserted reconstruction.

### Complications and failure

Of the seven patients, three patients had complications. Three patients (patients 1, 4 and 6) showed a dehiscence at the recipient site as a consequence of augmentation. In patient 1, the dehiscence (< 5 mm), which was located over the screw and a prominent bony edge, could fully recover after smoothening the bony edge as well as removing the screw. This patient had additional haematoma formation which was treated with intravenous antibiotic therapy. The dehiscence in patient 4 of 2 mm was self-limiting showing full recovery of the soft tissue after 1 month. Implant insertion in these two patients was not affected.

Patient 6 with a wide augmentation area (region 13–23) was treated with three bone blocks. One of them failed, as osseous integration was not successful. Additionally, the dehiscence mentioned above was observed in this case. The failing bone transplant was removed and re-augmented over a year later with a bone block of the zygomatic alveolar crest of the other side (right) with successful implantation thereafter.

In none of the cases was a paraesthesia of the infraorbital nerve or secondary bleeding observed.

Table 2 shows a summary of all complications and the treatment for each patient.

**Table 2:** Complications and treatment

Patient	Augmentation area	Nr. of blocks	Complications	Therapy
**1**	12	1	• Dehiscence over the screw/bony edge • Haematoma formation	• I.V. antibiotic therapy • Smoothening of the bone
**2**	11, 21	1	-	-
**3**	21, 22	1	-	-
**4**	15, 16	1	• Dehiscence of 2 mm mesially of tooth 17	• None, self-limiting
**5**	11, 12	1	-	-
**6**	13/12, 11/21, 22/23	3	• Failing osseous integration of one bone block (region 13) with a dehiscence	• Graft-removing • Re-augmentation in the same area
**7**	21–25	2	-	-

Table 2 shows the complications that occurred and the treatment that followed for each patient.

## Discussion

This retrospective study of onlay bone grafts harvested from the zygomatic alveolar crest in a staged augmentation procedure analysed the increase in bone volume, complication rate, and overall success. As current literature concerning the zygomatic alveolar crest as an intraoral donor site is rare, this study contributes to the analysis and evaluation of this procedure. The evaluation of volume gain in the present study shows the capacity of the zygomatic crest for bone augmentation.

Patients 6 and 7 both received multiple grafts from the same donor site, 3 and 2, respectively. The alveolar zygomatic crest also provides enough bone in cases where multiple areas need bone augmentation.

Complications included wound dehiscence, which occurred in three patients at the recipient site. One of those patients showed a palpable bone crest after surgery, which may have been the reason for the development of a soft tissue defect of < 5 mm (patient 1). Wound dehiscence decreased and fully recovered after bone smoothening and screw removal. In another case (patient 6), a small area of exposed bone was present prior to zygomatic bone augmentation, which precluded fully healthy and stable soft tissue circumstances. The dehiscence showed incomplete regression, leaving a small persistent area of exposed bone. In this case, the patient underwent a wide and complex augmentation in region 13–23 using three bone fragments from the left zygomatic alveolar crest. A possible reason for the dehiscence is the missing osseous integration of one of the bone fragments. This bone block was removed, and the area was re-augmented more than 1 year later with bone fragments from the right zygomatic alveolar crest, which succeeded and provided enough bone for subsequent implantation. In the third case, a small dehiscence healed spontaneously.

One of these patients (patient 1) additionally formed a haematoma. The patient required intravenous antibiotics, which led to the complete regression of swelling and pain. Only one patient was a smoker (22.5 pack years) and no complications were observed.

The outcomes and complications of autogenous bone harvesting of the zygomatic alveolar crest was evaluated by Sakkas et al. [12] in a large-scale clinical study (113 grafts), which had a complete success of 82.3%. Compared to the findings of the present study, their study showed complications such as dehiscence, wound infection, or swelling but had a lower complication rate of 17.7%. In the present study, no case of sinusitis or paraesthesia of the infraorbital nerve could be observed. Graft failure occurred in one of our patients; thus, our study shows a higher rate of complications than the study of Sakkas et al. [12] most likely due to significantly fewer cases.

The advantage of the zygomatic alveolar crest is its suitable convexity for reconstruction in the anterior maxillary region and its bone quality due to its cortical bone structure. Furthermore, there is a low morbidity of the donor site. Anatomically, no running muscle strands cover the harvest site [2, 10]. Sakkas et al. [12] stated in their clinical study that bone grafts from the zygomatic alveolar crest can be easily harvested due to its good accessibility and low complication rate. The limiting factors are the anatomical vicinity to the infraorbital foramen as well as the mucous membrane of the maxillary sinus [2, 10]. Generally, intraoral donor sites allow treatment under local anaesthesia due to their proximity to the recipient site [2].

Bone grafts harvested from the zygomatic alveolar crest offer sufficient amount of bone for alveolar defects around one to three implants [11, 14]. In a cadaver study by Kainulainen et al .[14], an average of 0.59 ml of bone could be harvested which is comparable to our results (0.54 ml). In another study by Kainulainen et al. [15], a mean amount of bone of 0.9 ml could be harvested. This was sufficient to reconstruct defects around two or three implants and is comparable to the amounts harvested from the retromolar region [16].

The mandibular symphysis and ramus are classical intraoral donor sites. For surgery of the maxilla, these donor sites imply a second operating area. With the zygomatic alveolar crest, a locoregional treatment in the maxilla can be provided. Compared to the maxillary tuberosity, another maxillary donor site, the zygomatic alveolar crest is favourable in terms of morphology, access, and bone quality. The convex anatomy of the zygomatic alveolar crest is favourable for horizontal augmentation. Furthermore, the tuberosity shows lower bone quality in comparison to other intraoral donor sites [17, 18]. Moreover, the tuberosity needs to have a sufficient size to be an option for a bone graft. Concerning possible complications, bone grafts harvested from the tuberosity could be advantageous, especially regarding the relatively high complication rate in our study [19].Good accessibility with low donor site morbidity was reported by Gellrich et al. [2] with a healing period of 4 months, who stated that the zygomatic alveolar crest is well suited for augmentation of locoregional defects, as harvesting sites show little morbidity.

The iliac crest is the most commonly used extraoral donor site for autogenous bone augmentation and has the advantage of providing large amounts of bone volume. Kilinc et al. [20] calculated the volume of cancellous and cortical bones that can be grafted from the iliac crest, and it differed greatly compared to the bone volume that can be harvested from the zygomatic alveolar crest. However, in small- to medium-sized maxillary defects, the zygomatic alveolar crest provides sufficient bone for transplantation. Furthermore, a disadvantage of bone grafting from the iliac crest is the necessity of general anaesthesia.

Apart from autogenous bone grafting, bone substitutes, such as allografts or xenografts, are an alternative for augmentation. Compared to autogenous bone grafting, bone substitutes do not lead to donor site morbidity and have an unlimited supply [4, 6]. Nevertheless, autogenous bone augmentation remains the gold standard, as it has the advantages of osteoconduction, possible osteoinduction, osteogenesis, and lack of immunological responses [5, 10].

Brainlab software enabled merging CBCT scans and the segmentation of specific areas. This allowed for an accurate assessment of volumes of the region of interest. Since only one surgeon operated on all patients in this study, a performance bias can be excluded.

A limitation of this study is the low number of patients. Due to the retrospective design of the study, X-ray imaging was not standardised, leaving variability in the CBCT scanners and the interval time between augmentation and post-operative imaging. In average, the post-operative 3D imaging was taken 75 days after augmentation. In patients 2 and 6, the radiological evaluation was earlier due to a dehiscence and the loss of a bone graft (patient 6) and due to the explicit desire of the patient, in spite of detailed information about possible risks (patient 2).

Additionally, the observation time was limited, which precluded the study of long-term bone graft resorptions. To resolve these issues, further studies with long-term observation and standardised radiological follow-up settings should be performed.

Gultekin et al. [21] compared the bone resorption rate of autogenous ramus block bone grafting with guided bone regeneration. The mean volume reduction for autogenous bone grafting was 7.20% ± 1.40%, showing less bone resorption than guided bone regeneration. Similar data for the resorption rate of the zygomatic alveolar crest are not yet available. Considering bone quality, the mandibular ramus provides primarily cortical bone, similar to the zygomatic alveolar crest. This is an advantage that can address resorption; however, it may increase the risk for non-integration of the bone block [21]. The success of graft transplantation is largely determined by the degree of revascularisation, which occurs more rapidly in corticocancellous bone grafts than in pure cortical grafts [22].

Given the advantages of the zygomatic alveolar crest, this bone is well suited as a donor site for bone augmentation in the anterior maxillary region. Low donor site morbidity, good access, and its suitable convexity make it a valuable choice for autogenous bone augmentation [12]. The amount of bone volume harvested at the zygomatic alveolar crest provides enough bone material for augmentation for small- to medium-sized defects [12]. Even though less bone material is obtained compared to mandibular donor sites, the advantages, namely good access and good bone quality, outweigh this drawback.

For a thorough evaluation of this technique, further investigation of the rate and behaviour of bone resorption alongside more clinical trials providing long-term data, are needed.

## Conclusions

This study demonstrates the reliable increase in bone volume using the zygomatic alveolar crest as an intraoral donor site for maxillary alveolar crest augmentation. In our study, despite the small number of patients, complications occurred. Nevertheless, we regard the zygomatic alveolar crest as a valid possibility for locoregional bone augmentation. Since this technique addresses the osseous dimension problems in the same quadrant of the jaw, it reduces the need for additional donor regions. Thus, it may further support the acceptance of autogenous techniques.

## Data Availability

The datasets supporting the conclusions of this article are included within the article.

## Abbreviations

2D: Two-dimensional
3D: Three-dimensional
CBCT: Cone beam-computer tomography
Nr.: Number
